# The cascade of HIV care in Russia, 2011–2013

**DOI:** 10.7448/IAS.17.4.19506

**Published:** 2014-11-02

**Authors:** Anastasia Pokrovskaya, Anna Popova, Natalia Ladnaya, Oleg Yurin

**Affiliations:** Central Scientific Research Institute of Epidemiology, Russian Federal AIDS Centre, Moscow, Russian Federation

## Abstract

**Introduction:**

The cascade of HIV care is one of the main tools to assess the individual and public health benefits of antiretroviral therapy (ART) and identify barriers of treatment as prevention (TasP) concept realization. We aimed to characterize the changes in engagement of HIV-positive persons in care in Russia during three years (2011–2013).

**Methods:**

We defined seven steps in the cascade of care framework: HIV infected (estimation data), HIV diagnosed, linked to HIV care, retained in HIV care, need ART, on ART and viral suppressed (VL < 1000 copies/mL during 12 month ART). Information was extracted from the Federal AIDS Centre database and from the national monitoring forms of Rospotrebnadzor from the beginning of 2011 to 31 December 2013.

**Results:**

Nearly 668,032 HIV-diagnosed Russian residents were alive by the end of 2013, which consisted 49% of the estimated 1,363,330 people living with HIV. Among the alive HIV-diagnosed patients, 516,403 (77%) were linked to care and 481,783 (72%) were retained. Of 163,822 (25% of HIV diagnosed) patients who were eligible for ART, 156,858 (96%) were on treatment while 127,054 (81%) had viral suppression. However, only 19% of HIV-diagnosed patients achieved viral suppression which is necessary to prevent viral transmission. We noted substantial improvements over time in the proportion of individuals on ART. The proportion of patients who received ART increased from 24% in 2011 to 34% in 2013. The most significant leakages of patients during three years were on steps: “HIV infected → HIV diagnosed” (loss −55% in 2011, −53% in 2012, and −51% in 2013), “HIV diagnosed → Linked to care” (−23% yearly) and “Retained in care → Need ART” (−76%, −70%, and −66%, respectively).

**Conclusion:**

The stages of HIV diagnosis and estimation of ART eligibility were the most vulnerable to leakage. Encouraging HIV testing and earlier ART initiation are needed to maximize the effects of TasP interventions and to contain the spread of HIV in Russia.

